# Collaboration between meteorology and public health: Predicting the dengue epidemic in Guangzhou, China, by meteorological parameters

**DOI:** 10.3389/fcimb.2022.881745

**Published:** 2022-08-09

**Authors:** Jing Chen, Rui-Lian Ding, Kang-Kang Liu, Hui Xiao, Gang Hu, Xiang Xiao, Qian Yue, Jia-Hai Lu, Yan Han, Jin Bu, Guang-Hui Dong, Yu Lin

**Affiliations:** ^1^ School of Atmospheric Sciences, Sun Yat-sen University, Zhuhai, China; ^2^ Institute of Tropical and Marine Meteorology, China Meteorological Administration, Guangzhou, China; ^3^ Hospital for Skin Diseases (Institute of Dermatology), Chinese Academy of Medical Sciences and Peking Union Medical College, Nanjing, China; ^4^ Department of Research Center for Medicine, the Eighth Affiliated Hospital, Sun Yat-Sen University, Shenzhen, China; ^5^ School of Agriculture, Sun Yat-sen University, Guangzhou, China; ^6^ Department of Geography, Hong Kong Baptist University, Hong Kong, China; ^7^ NMPA Key Laboratory for Quality Monitoring and Evaluation of Vaccines and Biological Products, Sun Yat-sen University, Guangzhou, China; ^8^ Guangzhou Key Laboratory of Environmental Pollution and Health Risk Assessment, Department of Occupational and Environmental Health, School of Public Health, Sun Yat-sen University, Guangzhou, China; ^9^ Guangzhou South China Biomedical Research Institute co., Ltd, Guangzhou, China; ^10^ Shenzhen Withsum Technology Limited, Shenzhen, China

**Keywords:** dengue fever, temporal characteristics, spatial distribution, meteorological parameter, Spearman correlation analysis, generalized additive models

## Abstract

**Background:**

Dengue has become an increasing public health threat around the world, and climate conditions have been identified as important factors affecting the transmission of dengue, so this study was aimed to establish a prediction model of dengue epidemic by meteorological methods.

**Methods:**

The dengue case information and meteorological data were collected from Guangdong Provincial Center for Disease Prevention and Control and Guangdong Meteorological Bureau, respectively. We used spatio-temporal analysis to characterize dengue epidemics. Spearman correlation analysis was used to analyze the correlation between lagged meteorological factors and dengue fever cases and determine the maximum lagged correlation coefficient of different meteorological factors. Then, Generalized Additive Models were used to analyze the non-linear influence of lagged meteorological factors on local dengue cases and to predict the number of local dengue cases under different weather conditions.

**Results:**

We described the temporal and spatial distribution characteristics of dengue fever cases and found that sporadic single or a small number of imported cases had a very slight influence on the dengue epidemic around. We further created a forecast model based on the comprehensive consideration of influence of lagged 42-day meteorological factors on local dengue cases, and the results showed that the forecast model has a forecast effect of 98.8%, which was verified by the actual incidence of dengue from 2005 to 2016 in Guangzhou.

**Conclusion:**

A forecast model for dengue epidemic was established with good forecast effects and may have a potential application in global dengue endemic areas after modification according to local meteorological conditions. High attention should be paid on sites with concentrated patients for the control of a dengue epidemic.

## 1 Introduction

Dengue virus is sensed and resisted *via* toll-like receptor recognition in keratinocytes (Wang and Li, 2020), which activate specific CD8 ^+^ T lymphocytes and transfers skin lymphocyte-associated antigen to the skin for expression through virus-specific CD4^+^ and CD5^+^ T lymphocyte-related circulation, causing clinical symptoms. Dengue has become an increasing public health threat around the world due to its high incidence and fast spread rate, serious health consequences including death, and lack of effective treatment and vaccine (Promprou et al., 2005). In the past 15 years, the global incidence of dengue fever has increased by 70%, with 390 million people suffering from dengue fever annually and involving 128 countries and regions (Bhatt et al., 2013).

In China, dengue cases have been reported every year since 1997, and dengue has become one of the critical infectious diseases to be prevented and controlled. Of the whole country, Guangzhou City is the hardest hit area of dengue with 90% cases reported. It may be related to the geographical and climatic environment of Guangzhou, which is located in south China and close to Southeast Asian countries. The dominant vector of dengue transmission in Guangzhou is *Aedes albopictus* (Liu et al., 2017), and aquatic habitats contain the highest concentration of larval mosquitoes in urban areas and the lowest concentration in exurban areas (Zhu et al., 2016).

In the history of Guangzhou, there were three occasions of which the number of cases in 1 year exceeded a thousand (2006, 2013, and 2014). One outbreak happened in 2014 and affected 20 cities, with over 42,335 cases reported (Liu et al., 2018; Chen et al., 2019). **Supplementary Figure S1** shows the geographic distribution of dengue cases in 2014, which is presented as an orange–red area denoting the concentration of a large number of cases. Guangzhou is the center of the Guangdong–Hong Kong–Macao Greater Bay Area and surrounded by Hong Kong, Shenzhen, Foshan, Zhuhai, and Macao, which means that the six core cities are facing the threat of dengue transmission. The government annually spends 200 million yuan on disease control, but the results are far from satisfying. With the deepening of regional cooperation in the urban agglomeration and the acceleration of population flow of Guangzhou and the surrounding areas, this Greater Bay Area is facing more severe challenges of dengue disease control than in 2014. Therefore, it is urgent to establish an early warning and prediction system with high precision for dengue epidemic.

Climate conditions have been identified as important factors affecting the transmission of dengue (Chien and Yu, 2014; Alkhaldy, 2017; Sanchez-Gonzalez et al., 2018)—for example, temperature affects the biting activity and the distribution of *Aedes aegypti* (Couret and Benedict, 2014) and virus development (Mutheneni et al., 2017). Humidity also has an impact on the population density of female mosquitoes. Furthermore, mosquitoes live longer and disperse further under high relative humidity conditions (Promprou et al., 2005). A distinct seasonal pattern in the outbreaks of dengue viruses around the world was consistent with the beginning and end of rainy season (Promprou et al., 2005). An increase in reported dengue cases was proved in association with an increase in average monthly rainfall and average monthly maximum temperature (Hu et al., 2012; Chien and Yu, 2014). The attempts to build predictive models from meteorological data began in 2001 (Schreiber, 2001), and several models were established, such as Generalized Cross-Validation (Sang et al., 2015) and Spatio-temporal Bayesian Hierarchical Model (Lowe et al., 2016).

Generalized Additive Models (GAMs) are commonly used to set a predictive model for infectious diseases (Hart et al., 2009; Gu et al., 2015; Ateba et al., 2020). Lin et al. (2016) used GAMs to assess the effectiveness of a community-based integrated intervention of dengue in Guangzhou and acheived significant effects to control outbreak in areas. While Li et al. (2017) established GAMs with only a 1-week lag based on Baidu Search Index data. Therefore, in this study, we aimed to develop tailored, climate-based forecasting models of dengue fever for Guangzhou City by GAMs with a longer forecast period and further investigated the influence of imported dengue cases on the disease epidemiology. This study took the collaboration between meteorology and public health one step further by co-developing a dengue early probabilistic forecast model using meteorological parameters, which could provide markedly improved forecasting techniques for disease prediction and could potentially be operationalized as a meteorological service for the public health sector.

## 2 Methods

### 2.1 Meteorological data

We collected the meteorological data from January 1, 2005 to December 31, 2016 from Guangdong Meteorological Bureau, including daily average temperature, daily precipitation, daily average humidity, and daily weather phenomena (days of continuous rainfall and continuous days without rainfall). The meteorological data analyzed in this article mainly came from Guangzhou National Weather Station. The station is no. 59287, and its latitude and longitude is 23°21′ N and 113°48′ E. It collects the meteorological data of Guangzhou City from 262 automatic weather stations.

### 2.2 Dengue case data

We collected the dengue case data between January 1, 2005 and December 31, 2016 from Guangdong Provincial Center for Disease Prevention and Control, including the number of cases reported per day with the onset time, the detailed address of the patients, and whether the case was imported.

Imported cases are defined as those who have traveled to dengue endemic regions and been bitten by mosquitoes less than 15 days before the symptom onset (Sang et al., 2015). The information of imported cases, including disease course information and demographic information, was provided by Guangdong Province Center for Disease Prevention and Control.

### 2.3 The interpolation of climatic data of dengue cases

The longitude and the latitude of automatic stations in Guangzhou City and the residential addresses of dengue cases were collected from Guangdong Meteorological Bureau and Guangdong Provincial Center for Disease Prevention and Control, respectively. The meteorological data of each dengue case were transformed according to the distance between the locations of cases and automatic stations, and the way for transformation is called interpolation (Belcher and Degaetano, 2007). We interpolated the meteorological data of the location of each dengue case at the date of diagnosis by **Formula 1** (Belcher and Degaetano, 2007), and the calculated meteorological data were used for the further analyses of the relationship between meteorology and dengue cases.

**Formula 1**


$$Z=\sum_{i-1}^{n}\frac{\frac{Z_{i}}{d_{i}^{2}}}{\sum_{i=1}^{n}\frac{1}{d_{i}^{2}}}$$

where *n* is the sample number, *Z_i_* is the climatic data of the sampling site, and *d_i_* is the distance from *i* to the interpolated site. When the sampling site and the interpolated site overlap, the sampling site weight is 1, and the *Z* value of the interpolated site is equal to that of the sampling site. Sampling site is the location of Automatic Weather Station with a fixed location and set by the Meteorological Bureau in the city. The interpolated site is the location of a dengue case.

### 2.4 Statistical analyses

Data were presented as mean ± SD, and the correlation between the number of dengue cases and the meteorological factors in epidemic was analyzed by Pearson correlation analysis (Tang et al, 2017), Spearman correlation analysis, and GAM models. A *P*-value of<0.05 was considered statistically significant.

All the statistical analyses were performed using the statistical software R 4.1.2 (R Foundation for Statistical Computing, Vienna, Austria).

#### 2.4.1 Spatio-temporal correlation analysis of imported dengue cases

According to the onset time information and residential address of the imported cases, a continuous time series was formed from January 1, 2005 to December 31, 2016. Then, high-precision geographic grid points were used to conduct a spatio-temporal correlation analysis of the imported cases by the following steps: if the onset time of a case was before entry to Guangzhou, the entry time was taken as the starting point for the analysis; if the onset time was after entry, the onset time was taken as the starting point. The density of dengue patients was calculated daily from the starting point.

The residential address of the imported case was taken as the center of the circle, and the density of patients in different radii was calculated within 20 km using 100 m apart. According to the flight distance of mosquitoes, it is generally believed that the range of a mosquito’s activity is mainly within 100 m of its birthplace, and the maximum is no more than 1 km (WHO, 2009). We mainly calculated the incidence of dengue in this area with high risk, and the 20-km distance evaluated is set to evaluate the impact of imported case as comprehensively as possible. The distance of non-imported case from the imported case was calculated according to **Formula 2**.

**Formula 2**: Distance calculation formula from *D_i_* to *D_i_* + 1:


$$\frac{\sum_{x=1}^{nx}\sum_{y=1}^{ny}H|L_{y}-L_{x}|}{ny*nx}\{\begin{array}{c} H|L_{y}-L_{x}|=1;D_{i}\leq |L_{y}-L_{x}|\leq D_{i+1}\\ H|L_{y}-L_{x}|=0;|L_{y}-L_{x}|\leq D_{i}orD_{i+1}<|L_{y}-L_{x} \end{array}$$

where *x* is the imported case, *y* is the non-imported case, *L_x_* is the location of the imported case, *L_y_* is the location of the non-imported case, *D* is the distance unit (100 m), *D_i_* is the i-th unit distance, *|L_y_–L_x_|* is the distance between two cases and calculated by latitude and longitude, and *H* means Heaviside step function.

#### 2.4.2 Spearman’s correlation coefficient

Spearman’s correlations were used to investigate the association between dengue cases and local meteorological data. The correlation coefficient of each meteorological parameter with different lag days was calculated, and the identified lag days of the maximum coefficient were used to establish the GAMs and applied to predict the number of cases.

#### 2.4.3 GAMs

GAMs were used to analyze the non-linear influence of meteorological factors on local dengue cases and predict the number of local dengue cases.

We got interpolations of various meteorological factors by **Formula 1**, and the interpolations of meteorological element data were substituted to the forecast (**Formula 3)** for a high-precision prediction of any grid point of Guangzhou City.

The prediction **Formula 3** is as follows:


$$g\left(\mu \right)=\beta_{0}+\sum^{}\beta_{i}X_{i}+\sum^{}S_{j}(X_{j})$$

In **Formula 3**, *g*(*μ*) denotes a link function that can select the corresponding link function according to the different statistical distributions of the dependent variable. Consistent with previous studies, the distribution of dengue cases in this study fits a Poisson distribution. Thus, the corresponding link function for the GAMs is ln(*y*). *β*_0_ is a constant term, *β_i_* (*X_i_*) represents the linear fitting function, and *S_i_* (*X_i_*) represents the nonlinear fitting function (smooth function), so the dependent variable *y* refers to the number of local cases from 2005 to 2016 [ln(local cases). The independent variable *X_i_* represents ln(cumulative local cases from previous days +1), cumulative imported cases from previous days, and *X_j_* represents temperature, precipitation, and wind with the lag day of maximum correlation coefficient.

The GAMs are selected according to the generalized cross-validation (GCV) score and R-square, with a better model corresponding to smaller GCV values and bigger R-square. R-square is expressed as the percentage of the explained variance, which is a quality measure of the model’s fit to the data.

First, we used Spearman’s correlation coefficient to find the factors with the highest correlation with the number of dengue cases, including daily average temperature, accumulated precipitation, daily average wind speed for lag days (1–168 days), cumulative number of local cases in previous days (1–168 days), and cumulative number of imported cases in previous days (1–168 days). Then, the daily average temperature, accumulated precipitation, and daily average wind speed of the lag days with the highest correlation were used as the nonlinear part, and the cumulative number of local cases in previous days and the cumulative number of imported cases in previous days with the highest correlation are used as the linear part, and the GAMs were established. Since the lag days of daily average temperature, accumulated precipitation, and daily average wind speed were adjusted to different days, cumulative number of local cases, cumulative number of imported cases in different previous days, *etc*., the best model was obtained according to the smallest GCV and largest R-square.

After that, sensitive analyses were applied to demonstrate that the results were robust, of which precipitation was replaced with relative humidity, and daily average temperature, accumulated precipitation, and daily average wind speed were replaced with different lag days, *etc*.

## 3 Results

### 3.1 Overall temporal and spatial distribution characteristics of dengue cases

**Figure 1:**
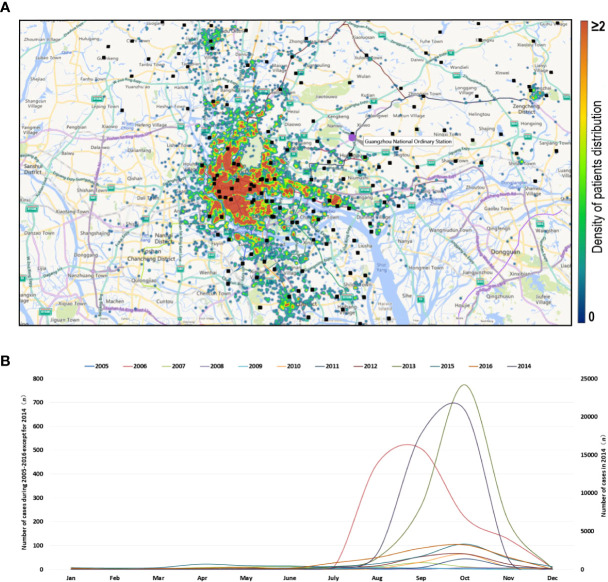
Temporal and spatial distribution characteristics of dengue cases from 2005 to 2016. **(A)** Accumulative spatial distribution of dengue cases from 2005 to 2016 in Guangzhou. Each patient with dengue fever reported from 2005 to 2016 represents a point, and all of the points are represented on the map. Black dots represent weather stations providing weather data. **(B)** Time distribution of dengue cases from 2005 to 2016. The left ordinate shows the case numbers of dengue for all years except 2014, and the right ordinate shows the case numbers of dengue in 2014.

**Figure 1A** shows the spatial distribution of patients during 1 January 2005 to 31 December 2016, and an obvious clustering was observed in Guangdong–Hong Kong–Macao Greater Bay Area.

From 1 January, 2005 to 31 December, 2016, the total number of dengue cases in Guangzhou was 46,206, with 343 imported cases. It is worth noting that 45,870 dengue cases were reported from July to November, accounting for 99.28% of the total cases. From January to June, only 335 cases (0.73%) were reported, and February had the lowest incidence during a whole year (**Figure 1B**).

Furthermore, 2014 had the most serious epidemic during the 12 years with 42,335 cases reported, accounting for 91.62% cases of the 12 years (**Figure 1B**), followed by 2006 (1,319 cases, 2.85%) and 2013 (1,311 cases, 2.84%). There were no more than 500 cases per year in the remaining 9 years.

### 3.2 Influence of imported cases on the epidemic of dengue

**Figure 2:**
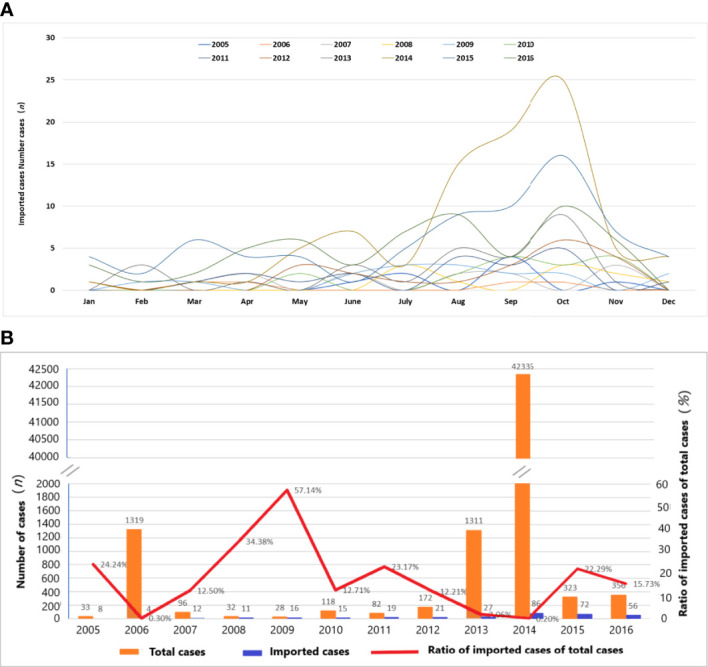
Temporal distribution characteristics of imported dengue cases from 2005 to 2016. **(A)** Time distribution of dengue imported cases. **(B)** Ratio of imported cases to total cases in each year.

From 1 January, 2005 to 31 December, 2016, 343 cases were imported, accounting for 0.74% of the total number of cases (**Figure 2A**). Among them, 86 cases were imported in 2014, followed by 2015 (72 cases) and 2016 (56 cases). **Figure 2B** shows the ratio of imported cases of the total cases in each year from 2005 to 2016. In some years, the ratio of imported cases was very high (2005, 24.24%; 2009, 57.14%; 2010, 23.17%; 2015, 22.29%; and 2016, 15.73%).

From May to November is the main period for imported cases. The number of imported cases reached 289, accounting for 83.29% of the total imported cases. The maximum number of imported cases was 80 in October, accounting for 23.05% of all the imported cases. The lowest import was in February, with a total of seven imported cases or 2.02% of the total imported cases. In the 12 years from 2005 to 2016, the first imported case occurred in January 1 at the earliest and in June 30 at the latest.

The Pearson correlation analysis shows that the correlation coefficient between the imported cases and the occurrence of dengue cases reached 0.891 under the confidence of *α* = 0.01, indicating that there was a positive correlation between the imported cases and the dengue incidence in Guangzhou.

**Figure 3:**
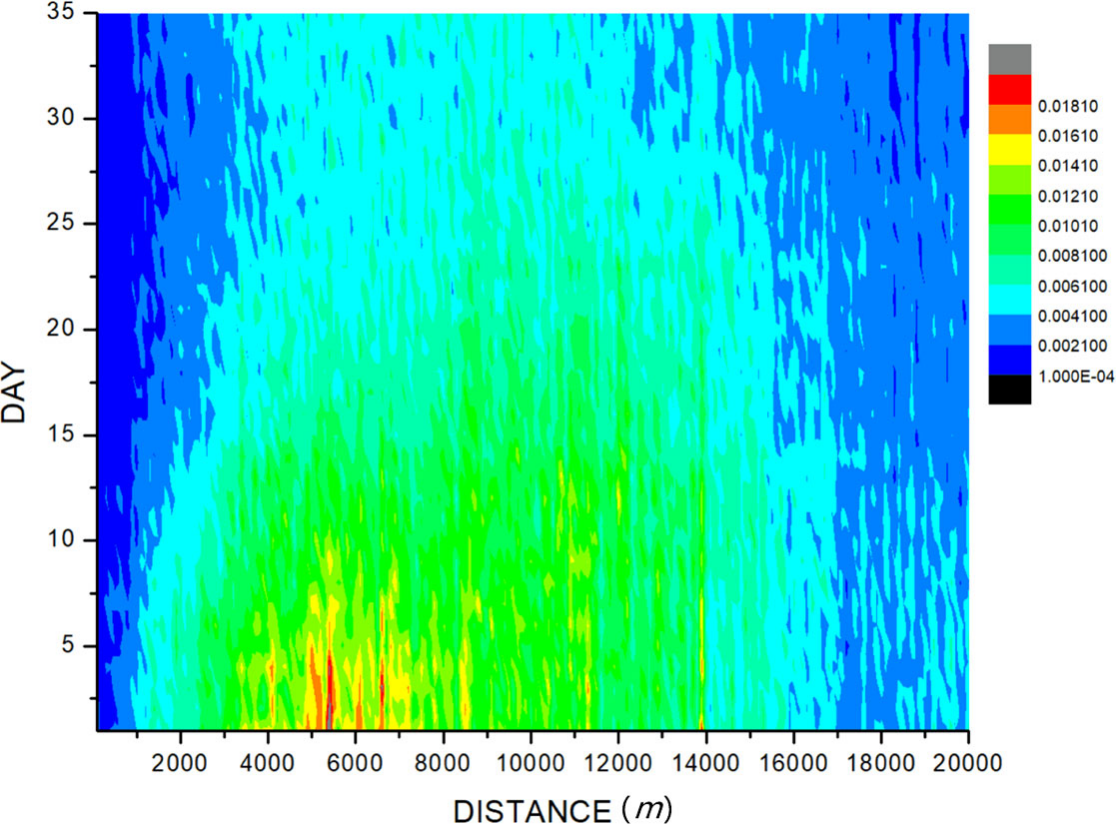
The influence of imported cases on the epidemic of surrounding dengue. The imported case was set as the original point, and the high-density area for case distribution is 5,000 to 6,500 m from the imported case on distance and within 5 days on time of diagnosis of the imported case, with a potential dengue incidence of 0.0181%. The area on the distance within 1,000 m and on time longer than 15 days shows a low-density area of cases reported, with a potential dengue incidence of only 0.0021%.

By **Formula 2**, the distance of new cases from the imported case was calculated. We set the imported case as the center; the horizontal axis was the distances from the case, and the vertical axis was the number of cases within different days after the onset time. The influence of imported cases on the epidemic of dengue cases around was evaluated in **Figure 3**. The results showed that the high-density area for case distribution is 5,000 to 6,500 m in distance and within 5 days in terms of time, with a potential dengue case incidence of 0.0181%. The spatio-temporal area at a distance within 1,000 m and of time longer than 15 days shows a low-density area of cases reported. It is generally believed that the range of the mosquito’s activity is mainly 100 m from its birthplace, and the maximum is no more than 1 km (WHO, 2009), and dengue incubation in the human body takes 3–10 days (Chan and Johansson, 2012), so the abovementioned spatio-temporal area is with most influence of the imported cases, but our result showed that the potential dengue case incidence is only 0.0021% (**Figure 3**).

### 3.3 Establishment of a dengue prediction model and verification of its forecast effects

#### 3.3.1 Establishment of a dengue prediction model

**Figure 4:**
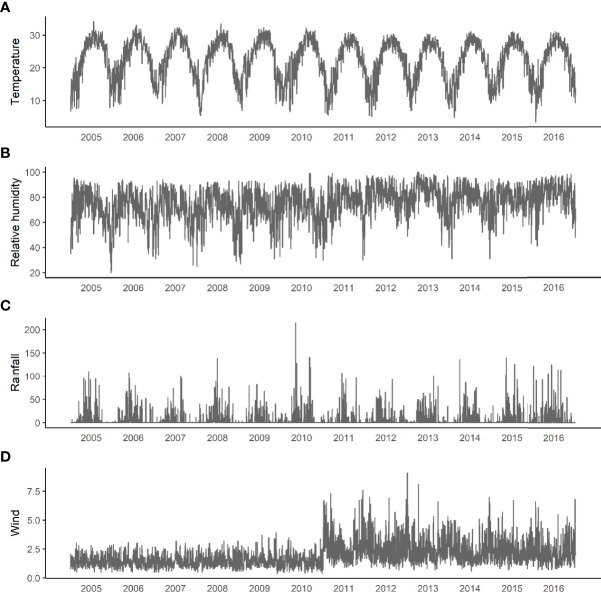
Changes in meteorological factors from 2005 to 2016. **(A)** Daily average temperature. **(B)** Relative humidity. **(C)** Accumulated precipitation. **(D)** Average daily wind.

We set Guangzhou as a grid point and plot meteorological parameters including temperature, relative humidity, precipitation, and wind speed of years 2005–2016 (**Figure 4**).

**Figure 5:**
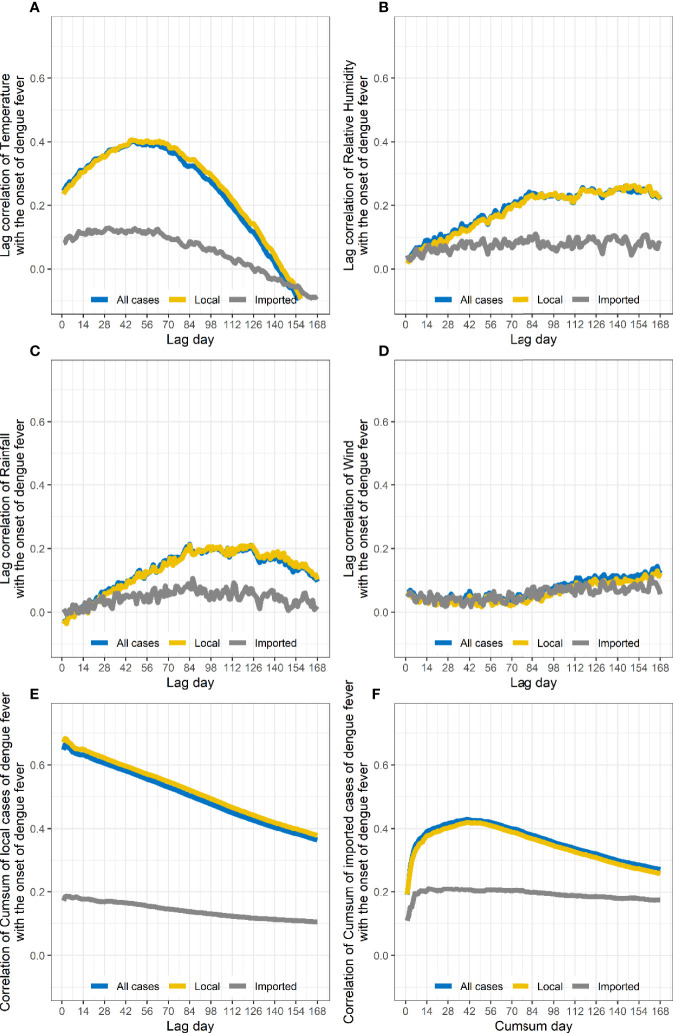
Changes in correlation coefficients between the number of people with dengue on different lag days and meteorological factor. The number of local cases is related to the daily average temperature **(A)**, relative humidity **(B)**, accumulated precipitation **(C)**, and average daily wind speed lagging 1–168 days **(D)** as well as the cumulative number of local cases in the previous 1–168 days **(E)** and the cumulative number of input cases in the previous 1–168 days **(F)**.

The correlation coefficient between the number of cases and the lag of meteorological parameters including temperature, precipitation, and wind speed for 1–168 days were calculated (**Figure 5**, all *P*-values<0.05). As relative humidity and precipitation are highly consistent, the final variables included in the GAMs were temperature, precipitation, and wind speed.

According to the Spearman’s correlation coefficient results, the meteorological conditions of lag 42 days, the cumulative number of local cases in the previous 2 days, and the cumulative number of imported cases in the previous 40 days were selected as the model’s adjust R-square = 0.989, deviance explained = 98.8%, and GCV = 0.970 (**Table 1**).

**Table 1:** Results of the Generalized Additive Models.

Parameter	Coefficient	Standard error	*t*-value	*P*-value
**Intercept**	-1.884	0.060	-31.36	<0.001
**ln(cumulative imported cases in previous 40 days + 1)**	1.095	0.006	185.68	<0.001
**Cumulative imported cases in previous 2 days**	-0.012	0.001	-11.32	<0.001
**S (temperature lagging 42 days)**	8.676	8.917	31.30	<0.001
**S (precipitation lagging 42 days)**	8.953	8.998	25.88	<0.001
**S (wind lagging 42 days)**	7.387	7.847	14.91	<0.001

#### 3.3.2 Verification of forecast effects of the dengue prediction model

We used the average meteorological data of Guangzhou to plot the incidence curve of dengue of years 2005–2016 by the Forecast and verified it by the actual incidence.

**Figure 6:**
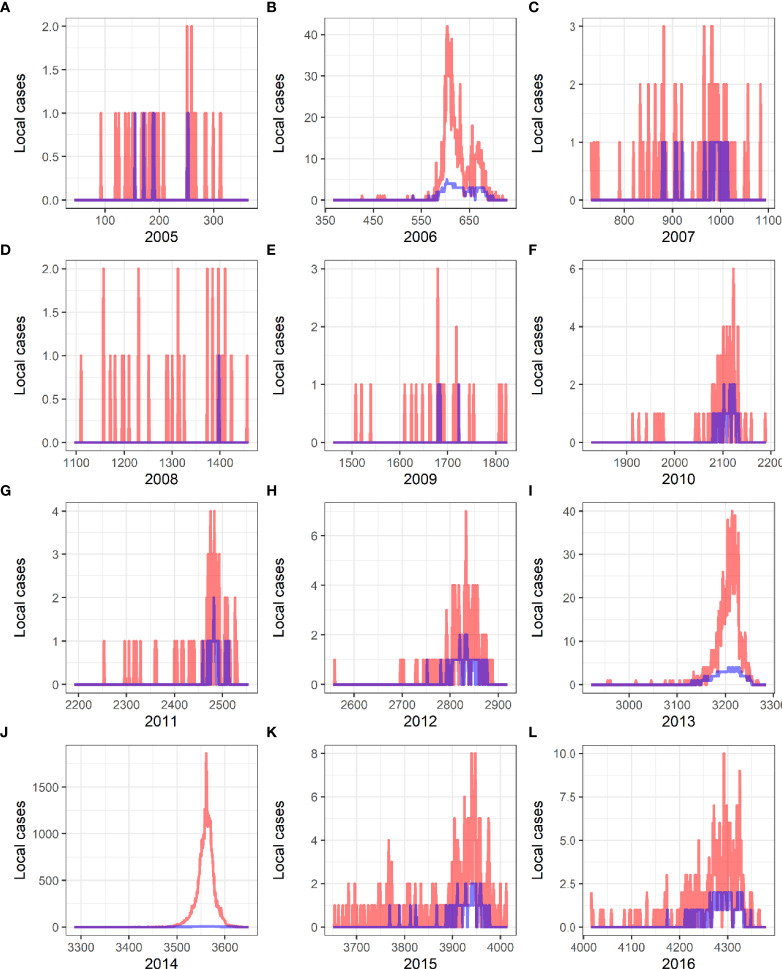
Verification of predictive results and actual incidence of local dengue fever from 2005 to 2016 in China. Red curves present the actual cases, and blue curves are the predicted curves created by Generalized Additive Models. **(A)** 2005. **(B)** 2006. **(C)** 2007. **(D)** 2008. **(E)** 2009. **(F)** 2010. **(G)** 2011. **(H)** 2012. **(I)** 2013. **(J)** 2014. **(K)** 2015. **(L)** 2016.

The curve of daily occurrence of dengue cases and the actual number of daily cases from 2005 to 2016 are shown in **Figure 6**, and the results showed that the predicted curve by the forecast model is in concordance with the actual case occurrence trend, with a forecast effect of 98.8%.

## 4 Discussion

In this study, we established a forecast model of dengue incidence by cooperation of meteorological and epidemiological data in Guangzhou City. The model can accurately predict dengue incidence and epidemiological status in Guangzhou 1.5 months in advance, which is superior to the Time Series Poisson Regression Analysis models (Lu et al., 2009; Sang et al., 2015).

Since the relationship between climate information and dengue has been confirmed (Promprou et al., 2005; Shang et al., 2010), the establishment of a forecast system for dengue epidemic becomes a research hotspot. Most of the studies on forecast system used statistical methods to produce dengue predictions. Jain et al. (2019) used GAMs to fit the relationships between the predictors and the clinical data of dengue from 2008 to 2012 in Thailand and concluded that the model allowed to make forecasts with a lead time of 1 month. Shi et al. (Shi et al., 2016) developed a set of statistical models using least absolute shrinkage and selection operator methods to forecast the weekly incidence of dengue notifications over a 3-month time period. Lowe et al. (2017) used seasonal climate and El Niño forecasts to predict the evolution of the dengue season in Machala, and the autoregressive model predicted dengue one calendar month in advance.

Differently from the abovementioned studies, which used statistical methods to obtain probabilistic forecasting equation by studying climate factors and dengue cases through correlation, the following studies set up dengue prediction models by meteorological parameters. Lowe et al. performed several studies on the relationship between dengue and meteorological factors in Brazil, based on which they established an early warning system driven by real-time seasonal climate forecasts and the dengue cases reported to the Brazilian Ministry of Health in February 2014 (Lowe et al., 2011; Lowe et al., 2014; Lowe et al., 2015; Lowe et al., 2016); however, they used incomplete surveillance data to drive the model, the spatial resolution of the forecasts was coarse, and information regarding the (re)introduction of different serotypes or vector control activities were lacking. Lee et al. (Lee et al., 2018) used a sensitivity analysis of the temperature-dependent parameters to explore the effects of climate change on dengue transmission dynamics. Yap et al. (Yip et al., 2022) used a rigorous Bayesian modeling framework to construct an early warning system for the central region of Malaysia and found 46.87% increase in dengue cases due to an increase of 1°C in the central equatorial Pacific sea surface temperature with a lag time of 6 weeks.

The current study weighed the influence of meteorological indexes on dengue transmission, set a forecast model by GAMs, and successfully confirmed that the predicted curve is in concordance with the actual case occurrence trend, with a forecast effect of 98.8%, which exceeded that of a Thailand study (73%) (Jain et al., 2019) using GAMs with the additional inclusion of socio-economic factors, of a Singapore study (95%) (Shi et al., 2016) using least absolute shrinkage and selection operator methods, and of an Ecuador study (90%) (Lowe et al., 2017) using a Bayesian hierarchical mixed model.

For the role of imported cases in dengue epidemic, we got some interesting results. The statistical analyses showed that the correlation coefficient between the imported cases and the occurrence of dengue reached 0.891 under the confidence level of *α* = 0.01, indicating that there was a positive correlation between the imported cases and dengue incidence. However, when we used the spatio-temporal method to further analyze the influence of the imported case on disease epidemic, we found that, around the high-risk area (1,000 meters) of the imported case within a period of 15 days, the potential dengue case incidence is only 0.0021%, which means a very slight influence of the imported case on the incidence of surrounding cases. The conclusion is inconsistent with a previous study (Peng et al., 2012), in which the authors concluded that the 2014 dengue outbreak in Guangzhou was initiated by an imported case from Southeast Asia based on the epidemiological results. We also found that the influence of imported cases on annual epidemic is closed to zero, even in the years with a high ratio of imported cases [2005, 8/33 (24.24%); 2009, 16/28 (57.14%); 2011, 19/82 (23.17%); 2015, 72/323 (22.29%); and 2016, 56/356 (15.73%)—all of these years had a total number of cases less than 500. Thus, combined with the abovementioned information, we concluded that, from 2005 to 2016, the sporadic single or the small number of imported cases of dengue may have a very slight effect on the area around where the patient lives and have not caused an epidemic of dengue around.

Zhao et al. (2020) reported that the 2014 dengue outbreak was probably triggered by a new strain imported from other regions, and a new strain most likely invaded Guangdong in April 2014. The results of the dynamics of dengue infectivity were consistent with our results as lag of 42 days plus incubation period for dengue fever (1 to 2 weeks), which results in a disease break in the summer season because of April import. We speculate that the imported cases may be clustered multiple cases.

The study has the following limitations: (1) The study directly collected the reported dengue cases from the local Disease Prevention and Control Department, but people who caught dengue with few or no symptoms were missed. (2) The study did not include other socio-economic factors, which may have an influence on its objectivity. Despite these limitations, this work advances the state-of-the-art of climate services for the public health. The current dengue prediction model can predict a dengue epidemic in Guangzhou 1.5 months in advance, which significantly aids in the management of scarce medical resources by the responsible institutions. However, the efficacy of the current meteorology­based dengue prediction model highly depends on the availability of accurate climate information, which varies very much among regions. Meanwhile, the living habits of *Aedes* mosquitoes are impacted by current climates—for example, the biting activity of *A. albopictus* varies from place to place, such that the activity peak in Macau (near Guangzhou) is observed between 06:00–08:00 and 18:00–20:00 (Almeida et al., 2005), but in India, it shifts to 22:30–23:00 and 20:30–21:00 (Sumodan, 2014). The dengue vector is *Aedes aegypti*, not *A. albopictus*, in many countries, including Queensland, Australia (Tun-Lin et al., 2000), Thailand, Puerto Rico (Scott et al., 2000), and Bangladesh (Zahirul Islam et al., 2018). The influence of climates on different kinds of *Aedes* mosquitoes needs to be investigated. The decision of the 42-day lag as a parameter in the prediction model is based on the association between the meteorological data and the incidence and epidemic of dengue fever, including the impact of climate on the vector. When these abovementioned conditions change, the model will inevitably change, so the current dengue prediction model established based on the precise meteorological data of Guangzhou is only recommended to be applied to the local city. The successful implementation of the current prediction model servicing for health in various regions depends on the availability of relevant, high-quality meteorology data as well as the capacity to transform the data into reliable and tailored climate products, which relies on the close collaboration between public health specialists, climate scientists, and mathematical modelers.

In conclusion, we successfully established a Probabilistic Forecast Model of dengue epidemic including the number and density of incidence using meteorological factors with a good forecast effect, which was verified by the actual incidence data of dengue in Guangzhou from 2005 to 2016. The study provides evidence for the Public Health Department to generate a strategy for the prevention and control of dengue. The current Probabilistic Forecast Model is only for Guangzhou City because of the restriction of limited meteorological data, while the way to establish a valid Probabilistic Forecast Model of dengue by local meteorological data is worthy to be promoted among dengue endemic areas of the world. Additionally, we confirmed that sporadic single or a small number of imported cases have a very slight influence on the dengue epidemic around, so high attention should be paid on the sites with concentrated patients. Thus, the prevention and control policies should be regulated according to the new evidence.

## Data availability statement

The data analyzed in this study is subject to the following licenses/restrictions: The data of dengue cases came from the Guangdong Provincial Center for Disease Prevention and Control, and the meteorological data of the same period came from the Guangdong Meteorological Bureau. The data that support the findings of this study are available from JB (dr.jinbu@gmail.com) upon reasonable request. Requests to access these datasets should be directed to JB (dr.jinbu@gmail.com).

## Ethics statement

Ethical approval/written informed consent was not required for the study of animals/human participants in accordance with the local legislation and institutional requirements.

## Author contributions

JB, GH-D, and YL provided overall guidance and managed the project. JC set up the meteorological model. YL and RL-D set up the statistical model of the data. JB finished the manuscript on the basis of comments from other authors. All other authors provided data, developed models, reviewed results, provided guidance on methods, or reviewed the manuscript. All authors contributed to the article and approved the submitted version.

## Funding

This work was supported by the National Natural Science Foundation of China (82003418), the National Key Research and Development Project (2018YFE0208000), and Science and Technology Planning Project of Guangdong Province (2021B1212020016).

## Conflict of interest

Author YL was employed by the company Guangzhou South China Biomedical Research Institute co., Ltd. and Shenzhen Withsum Technology Limited.

The remaining authors declare that the research was conducted in the absence of any commercial or financial relationships that could be constructed as a potential conflict of interest.

## Publisher’s note

All claims expressed in this article are solely those of the authors and do not necessarily represent those of their affiliated organizations, or those of the publisher, the editors and the reviewers. Any product that may be evaluated in this article, or claim that may be made by its manufacturer, is not guaranteed or endorsed by the publisher.

## Supplementary material

Supplementary Figure 1 The geographic distribution of dengue cases in 2014. Each patient with dengue fever reported from 2005 to 2016 represents a point, and all of the points are represented on the map. The patients with dengue fever are concentrated in the Guangdong-Hong Kong-Macao Greater Bay Area, covering an orange red area.
