# Supplementary figures and images for: Collaboration between meteorology and public health: Predicting the dengue epidemic in Guangzhou, China, by meteorological parameters
Source: Front Cell Infect Microbiol. 2022 Aug 9;12:881745. doi: 10.3389/fcimb.2022.881745 (PMC9397942; doi:10.3389/fcimb.2022.881745)

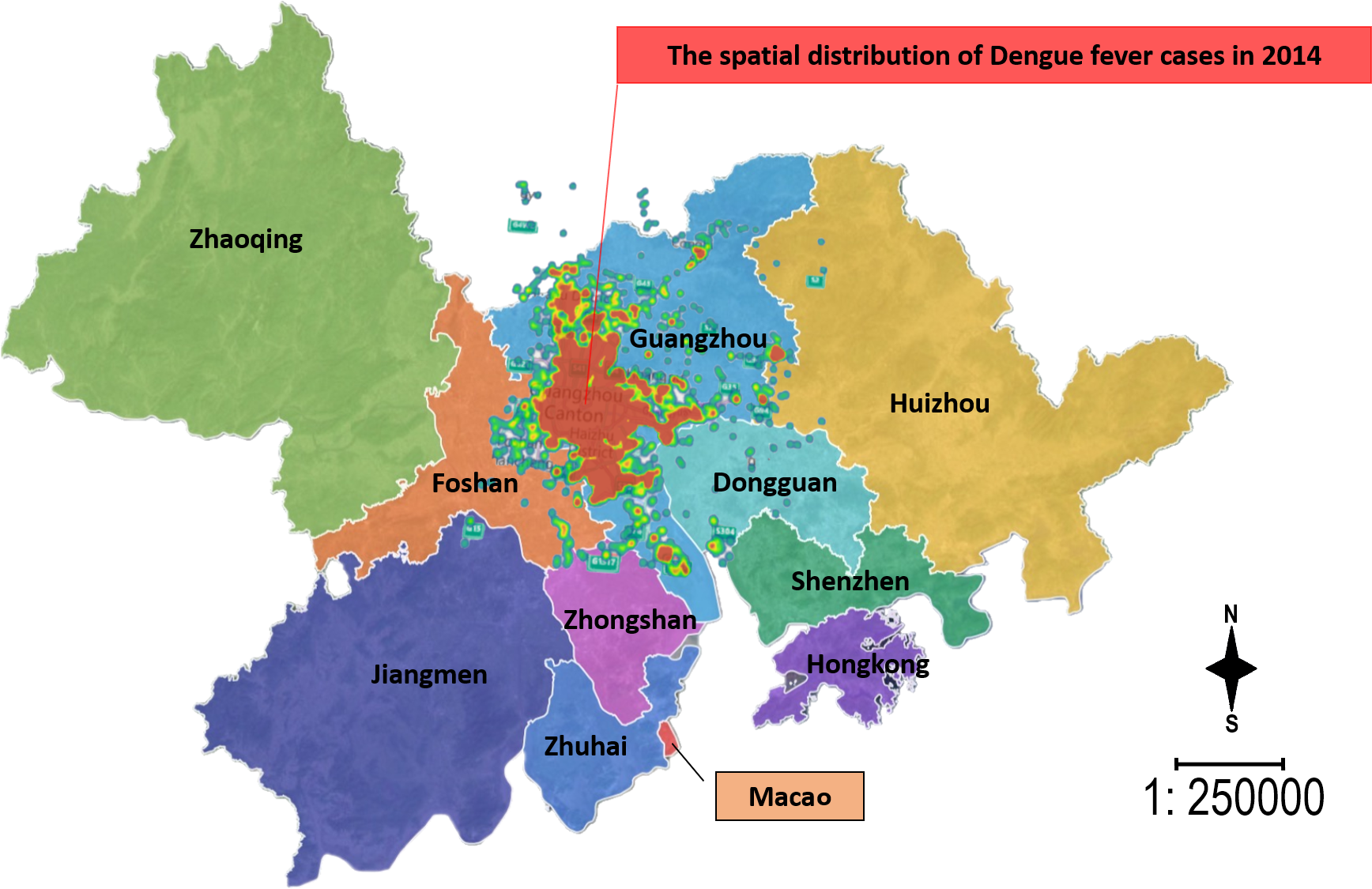

Supplement: Supplementary Figure 1 — The geographic distribution of dengue cases in 2014. Each patient with dengue fever reported from 2005 to 2016 represents a point, and all of the points are represented on the map. The patients with dengue fever are concentrated in the Guangdong-Hong Kong-Macao Greater Bay Area, covering an orange red area. [file Image_1.tiff]
